# Leveraging Neuroscience to Fight Stigma Around Mental Health

**DOI:** 10.3389/fnbeh.2021.812184

**Published:** 2022-02-28

**Authors:** Osborne F. X. Almeida, Nuno Sousa

**Affiliations:** ^1^School of Medicine, University of Minho, Braga, Portugal; ^2^Max Planck Institute of Psychiatry, Munich, Germany

**Keywords:** public stigma, self stigma, mental health, discrimination, social neuroscience

## Abstract

Labels serve as identifiers and convenient descriptors of inanimate and animate objects. In humans, given labels can easily become part of an individual’s self-perceived identity. Negative labels ascribed to a person can result in *internalized stigma*, a state that will shape the subject’s biography. This can ultimately impact the person’s mental and physical health since *perceived* and/or *anticipated stigma* discourages the use of social and health services. Per definition, stigma involves labeling of persons with physical, mental, or social characteristics that do not match the observer’s arbitrarily conditioned and calibrated sense of norms (*public stigma*); such labeling may eventually become embedded in rules, regulations, and laws (*structural stigma*). Internalized stigma projects onto a person’s emotions and actions. *Public (enacted) stigma* results from stereotyping (collectively agreed-upon notions about a group of persons that are used to categorize these people) and devaluation, which subsequently leads to social distancing, discrimination, and blatant abuse of human rights. Much of what we know about stigma results from research in the psychosocial sciences and, more recently, from social neuroscience. The stigma around mental health has generated much attention in the field of psychiatry where, to date, most research has focussed on epidemiology and anti-stigma interventions. This essay intends to stimulate thought, debate, and research within the behavioral neuroscience community and, therefore, to inform evidence-based design and implementation of neuroscience-based approaches by other professionals working towards the elimination of the stigma attached to mental illness. The article starts by considering the concept of stigma and the psychological processes that give rise to the phenomenon; it also considers how projected and perceived stigma are multiplied. Finally, after a brief review of the few existing neuroscientific explorations of stigma, gaps in our knowledge of the neurobiological basis of stigma are identified and discussed.

## About Stigma, Stigmatizing Attitudes and Stigmatization^1^

Stigma is a multifaceted concept, defined in the Merriam-Webster dictionary as “a mark of shame or discredit”. The word stigma originated in ancient Greece where slaves and other undesirables were branded to leave a mark (στίγμα) or “badge of dishonor”.

Erving Goffman’s work (1963) deserves credit for bringing stigma to the attention of behavioral scientists, referring to it as the reduction of a person “in our minds from a whole and usual person to a tainted, discounted one” or briefly, a person with “spoiled social identity” (Goffman, 1963). Stigma encompasses social, cultural, and moral processes (Kleinman and Hall-Clifford, 2009). Lauber’s (2008) definition of stigma as “a severe disapproval due to believed or actual individual characteristics, beliefs or behaviors that are against norms, be they economic, political, cultural or social”, serves as a good working definition of the phenomenon. Accordingly, stigma is rooted in the concept of distinguishing between “self” and “other” (Decety and Sommerville, 2003); such a distinction may also underpin self-stigma, a phenomenon in which stigmatized individuals consider themselves less worthy than others. In a classical piece of work, Patrick W. Corrigan dissected stigma into three components (stereotyping, prejudice and discrimination) that are manifest in a sequential manner (Corrigan, 2000).

Stigma may be expressed both subtly and overtly, for example, by avoiding direct eye contact, ascribing derogatory labels and names, and social distancing (avoiding contact, discouraging approaches). Subtle, or implicit, forms of stigma automatically trigger affective responses that reflect attitudes and biases that are innate, acquired or primed by an individual’s environment. In contrast to implicit biases, explicit biases occur within conscious awareness, are self-reportable, and regulatable through cognitive control processes (Stull et al., 2013; Comte et al., 2016). Briefly, explicit stigma operates through reflective (decisions based on factual knowledge and values), whereas implicit stigma is predominantly based on associative links and motivational orientations and is usually impulsive in nature (Nosek et al., 2011). Notably, implicit stigma is often a powerful predictor of stigmatizing attitudes and actions (Nosek et al., 2011; Scheff, 2014).

Stigma is a ubiquitous, pervasive, and cross-sectoral phenomenon. Everyone is likely to have a biased attitude that risks stigmatizing other individuals, groups, or organizations (Table 1). Much like in bygone centuries, stigma confers power to those who enact it. Stigma may also embrace a type of defensive reaction based on an (innate) fear of individuals different from the self; the latter belong to a so-called “out-group”. The norms used in both power- and fear-based behaviors give rise to attitudes (stereotype-based prejudice) and behavior (discrimination) that are stigmatizing.

**Table 1:** Characteristics of a person, group or organization that may kindle or trigger stigmatizing attitudes, reactions, and practices by those they encounter.

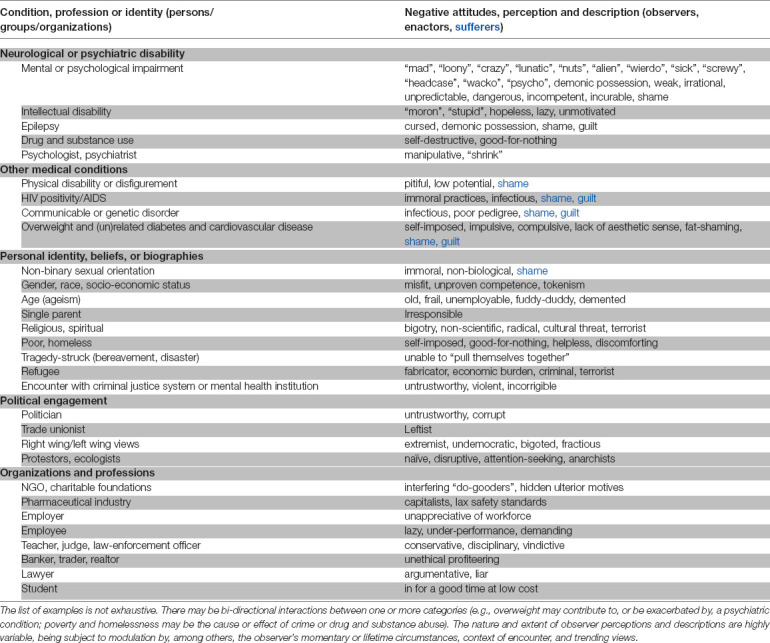

Interestingly, stigmatized persons may hold stigmatizing attitudes towards themselves (self-prejudice) or others with comparable or distinct “negative attributes”. Such attitudes may force them to conceal their condition (Bharadwaj et al., 2017) and to avoid approaches to service providers (World Health Organization., 2001,^2^; Stuart et al., 2012; Corrigan et al., 2014; Tsai et al., 2019). This is sometimes compounded by the paradoxical implicit and explicit stigmatizing attitudes held by professional caregivers (nurses, doctors, social workers; Lauber et al., 2006; Gaebel et al., 2011; Stuart et al., 2012; Henderson et al., 2014; Lebowitz and Ahn, 2014; Oliveira et al., 2020) towards the very people they are supposed to help and treat (Gaebel et al., 2015). Stigma held by professionals is usually reflected in physician-patient communication, optimism regarding treatment options, and prediction of the chance of recovery (see Peris et al., 2008; Wahl, 2012; Reihl et al., 2015; Haque et al., 2021). Interestingly, Loch et al. (2013) concluded that psychiatrists become increasingly stigmatized towards their patients as they become more familiar with the clinical features of the illness they are treating (also see Weiner et al., 1988). In addition, something that is often overlooked is that physicians (Haque et al., 2021) and other health professionals (Nyblade et al., 2019) may be burdened by their own somatic or mental health problems that are subject to stigma and, therefore, may be compromised in their diagnostic and care-giving functions. Importantly, the burden of stigma may extend beyond the primary object of discrimination (the afflicted person) to the patient’s social network of family, friends, and colleagues. This can be a barrier to holistic anamnesis since it excludes information provided by those familiar with the patient. Coupled with this, stigma at organizational and structural levels, undermines efforts to reduce health inequalities (Hatzenbuehler et al., 2013; Brewis and Wutich, 2019).

Family stigma (also called “courtesy stigma” when it extends to others with close contact with an out-group person) is another common form of stigma. This form of stigma is based on association alone. Family members are often blamed for inadequate nurturing skills or for the transmission of genetic flaws. Often, they are excluded from social relationships because of their likely “infectious” state. Thus, the cycle of stigma grows, with the development of internalized stigma among family and other close associates of the primary target of stigma, and vicarious stigma, where familiar bystanders suffer sadness or helplessness because of witnessing injustice, is often overlooked (Corrigan and Miller, 2004).

There are two, not necessarily mutually exclusive, working models that seek to explain stigma. The social cognitive model considers that stigma emerges from three sequential processes: stereotyping (negative beliefs about a group), prejudice (agreement with stereotyped beliefs and/or negative emotional reactions), and discrimination (behavioral consequences of prejudice such as exclusion from social and other opportunities) (Corrigan, 2000). Self-stigma also fits within this general framework: by accepting their label, individuals tend not to counter prejudices against them, which ultimately leads to adverse responses to their condition, including low self-esteem and -efficacy (Corrigan et al., 2009). On the other hand, the sociological model proposed by Link and Phelan (2001) centers around the two ideas that, (i) stigma is a societal force in which labeling acts in concert with the processes of stereotyping, separation, status loss, and discrimination; and (ii) interpersonal relationships are socially constructed.

As a phenomenon, stigma clearly has many aspects. As a subject of investigation, it is highly complex, not to mention the specialized language and terminology used in the psychological and social sciences (Link and Phelan, 2001; also see Sheehan et al., 2017 and Tsai et al., 2019). Without intending to oversimplify the challenge for understanding the biological basis of stigma, neuroscientists entering this domain of research initially need to grasp two, somewhat related, conceptual frameworks, namely, (i) that stigma embodies problems of **knowledge** (ignorance), **attitude** (prejudice), and **behavior** (discrimination; see Thornicroft et al., 2007); and (ii) that stigma emerges at three levels: **cognitive**, **emotional**, and **behavioral** (see Rössler, 2016).

Unsurprisingly, emotions lie at the core of stigma: on the one hand, disapproval, fear or reflexive disgust are emotions expressed by those projecting stigmatizing attitudes and behaviors, and on the other, stigmatized individuals feel ashamed of their condition. As discussed by Hatzenbuehler et al. (2009) and Burton et al. (2018), the experience of stigma is a form of (chronic) psychological stress. The stigmatized subject subsequently uses strategies of emotional regulation to cope with the stress. One of these strategies, attentional deployment, involves either a shift of attention away from, or a focus on, the stressful circumstance. Passive and repetitive focus on the problem (also referred to as rumination) worsens the emotional distress and may lead to depression (Hatzenbuehler et al., 2009; Miranda et al., 2013; Burton et al., 2018); therefore, rumination is a maladaptive strategy for dealing with psychological stress. Likewise, thought suppression (redirection of attention to other content) is considered maladaptive as it may impair memory (Richards, 2004) or lead to obsessive compulsive disorder (Ferreira et al., 2020). Concealment of a stigmatizing condition, albeit not immediately related to emotional regulation, is another non-productive strategy since it is associated with greater psychological distress (Quinn et al., 2014). Cognitive reappraisal (the process of altering how one thinks about an emotion-eliciting event such that the outcome is positive, or that the event carries less personal relevance; Gross, 1988) is a seemingly useful emotion regulation stratagem; however, the success of this approach seems to depend on the extent of control that the person facing stigma has over their own emotions vs. those of others (Troy et al., 2013). Given that so much current research in neuroscience is focussed on the feed-forward and -backward loops that regulate cognition, emotion and behavior, as well as their regulation by stress, examination of the neural basis of stigma is a potentially exciting addition to behavioral neuroscience research.

## Roots and Multipliers of Stigma

Like other types of behavior, stigmatizing behavior may be innate or acquired. Since comparator functions of the brain must play a role in determining action, it is reasonable to expect that stigma results from such built-in mechanisms although there is presently no direct evidence for this. In contrast, the social science literature indicates that cultural, spiritual, and animist beliefs and folklore are a key source of acquired stigma. Evolved over centuries of civilization, and still evolving, cultural beliefs are undeniably anchored in societies across the globe, although the weighting assigned to them may vary between individuals, groups, and locality. Moreover, they may be differentially evoked in different contexts, spiralling under the influence of popular trends and misinformation, out of feelings of solidarity, or what may be described as defensive herd-like behavior.

The environment plays an important role in shaping stigmatizing attitudes and ultimately, in enacting them. Importantly, stigmatizing attitudes may become entrenched during early life, as proposed by Scheff (2014). An analysis of children’s television films and cartoons in New Zealand revealed that characters portrayed as having a mental issue were frequent targets of disrespectful and negative vocabulary that implied that the character lacks control over their behavior (Wilson et al., 2000). From such portrayals, children implicitly learn that such language is acceptable and funny, and may separate, alienate, or put others down by bullying, intimidation or verbal harassment (see Rose et al., 2007). The stereotypes described by Wilson et al. (2000) increase the probability for generalizations about any mental condition and without insight into the suffering experienced by mentally ill persons. Negative depictions found in children’s programs are also found in the way persons with mental illness are represented (simple, childlike) in adult viewing programs (Wilson et al., 1999).

Print and other forms of media also play an undeniably important role in nurturing the general public’s stigmatizing views of mental illness: news is made more sensational by focussing on the danger and damage caused by mentally ill persons, rather than on the underlying causes of their illness (Angermeyer and Schulze, 2001; Corrigan et al., 2005). Since the public in industrialized countries has relatively easy access to information regarding mental health, including the biological underpinnings of mental health and illness (Pescosolido et al., 2010), it is striking that public acceptance of persons with psychiatric conditions either declined or remained unchanged in the period from 1990 to 2006 in wealthier countries (Schomerus et al., 2012). Moreover, an analysis of attitudes toward, and perceptions of, diabetes and schizophrenia expressed *via* social media platforms revealed that tweets about schizophrenia tended to be less medically accurate and more likely to be sarcastic and negative in tone than those about diabetes (Joseph et al., 2015)^3^. On a more positive note, however, a recent study reported a trend towards less stigmatizing coverage of mental illness in the print media and increased internet-facilitated mental health literacy (chiefly with respect to treatment options and stories of recovery) among the public (Hildersley et al., 2020).

Ignorance of the biological basis of physical and (especially) mental health ailments is a major trigger of stigma. Ignorance reinforces and perpetuates beliefs and hearsay about the causes of illness, and therefore boosts stigmatization but surprisingly, informing the public about the biological correlates of mental disorders (Schomerus et al., 2012; Loughman and Haslam, 2018; Lebowitz, 2019; Walsh and Foster, 2021) does not seem to be an effective measure against stigma around mental health. As noted in the previous paragraph, stigma prevails even in societies with access to education and access to information about mental illness. The question of whether the elicitation, mediation or execution of biased attitudes and stigmatizing actions might have a biological basis is addressed later in this article.

While toxic interactions between cultural factors and the lack of objective knowledge may be easily predicted and explained, there is a gap in knowledge of the extent to which single and repeated exposures to persons considered to have “negative” attributes contributes to stigmatizing behavior. Each exposure might be expected to reinforce pre-existing ideas (and memories) about a particular condition. If so, is it possible to interfere or erase such memories?

## Treatment Gaps Feed Cycles of Stigma Associated with Mental Health

Of all states, poor mental health appears to be the most stigmatized, with 116 of these descriptors were highly derogatory (Rose et al., 2007). This is striking because unlike many of the states/conditions listed in Table 1, persons with mental health issues do not usually display overt signs recognizable to the regular observer. The stigma associated with mental health—from personal to structural levels—is responsible for the generally poor treatment and rehabilitative arrangements for persons with psychiatric issues; the global scale of neglect of persons with psychiatric illness has been summed up as a “failure of humanity” (Kleinman, 2009).

Space limitations do not allow coverage of the large and growing literature that explores stigma, its negative impact on mental health outcomes, and stigma-reducing interventions. Excellent overviews of the subject can be found in expert-authored and -edited books (Thornicroft, 2006; Stuart et al., 2012; Gaebel et al., 2017). We here consider only a few selected aspects of the problem.

Epidemiological research indicates that around 40% of the general population receive a lifetime diagnosis of a mental illness. However, the treatment gap (or mhGAP, as it is called by the World Health Organization) is high in low-income countries where just 10% of the population have access to adequate diagnosis and care (Sweetland et al., 2014). This gap takes a high toll on the quality of life of individuals who are directly or indirectly affected by a neuropsychiatric condition^4^; it also impinges on community harmony and the economic prosperity of whole nations because of lost human potential and diversion of resources to literally “managing and containing” citizens suffering from mental illnesses.

Gone may be the days when mental patients were committed to asylums in the industrialized world (although solitary confinement of prisoners with psychiatric histories is still practised in some economically developed countries). On the other hand, asylum-like institutions exist in many poorer countries, partly reflecting gaps in treatment and neglect of human rights. Here, it is worthwhile noting that treatment outcomes for many psychiatric conditions are often better, or at least not worse when patients attend outpatient clinics, complemented by support from their communities than when they are hospitalized (Driessen et al., 2019). Notwithstanding the fact that hospitalization is the only (and best) option for some patients (e.g., those with severe episodes of disease, or who cannot be adequately served by existing outpatient and community care, may be a potential danger to self or others, live alone, have other comorbidities, or who are unlikely to comply with treatment regimens), unnecessary admissions to institutions only fuel stigma by suggesting that persons with mental health issues are “beyond help”. Further, hospitalized patients are often socially excluded, may be forced to engage in anti-social behavior or crime, lose dignity and educational and employment opportunities, and face disrupted family networks, while their families are shunned and thrown into emotional and financial despair (Bhugra et al., 2016). Besides the inestimable costs of poor mental health to individuals’ quality of life, mental illness takes a heavy toll on national budgets: in 2004, mental illness cost 25 countries of the European Union, Norway, Iceland and Switzerland an estimated €386 billion; 80% of these costs resulted from lost productivity (Andlin-Sobocki et al., 2005). Lastly, considering that mental and physical health problems frequently co-exist and that mentally ill persons may be denied access to treatment for physical comorbidities due to the stigmatizing attitudes held by some service providers, the true mhGAP and monetary cost may be much higher than estimated.

## What Might Neuroscience Research Contribute to Understanding The Biological Foundations of Stigma? Could Such Understanding Help Mitigate Stigma Around Mental Health?

Much research in behavioral neuroscience is done on the premise that increased understanding of the biological substrates and mechanisms that underlie a specific (abnormal or undesired) behavior will contribute to the design of tools that may be used to modify the expression of that behavior. Before considering some of the ways in which neuroscientific research on stigmatizing behavior could help reduce stigma, it is important to mention that a growing amount of evidence indicates that knowledge-attitudes-behavior practice (KABP) may be counter-productive in the fight against mental health-related stigma (Loughman and Haslam, 2018; Lebowitz, 2019; Walsh and Foster, 2021). It appears that, overall, while laypersons endorse neurobiological and genetic explanations of mental illness and may ascribe less blame to affected individuals for their problems, biogenetic explanations neither reduce the perception of the mentally ill as less dangerous nor reduce the social distance between healthy individuals and those suffering from mental illness (Haslam and Kvaale, 2015).

Despite the above caveat, we suggest that an understanding of the neural pathways and mechanisms that underpin the development of stigmatizing attitudes and their enactment could contribute to an evidence base that would support the development of psychosocial interventions that exploit brain plasticity to elicit behaviors such as adaptive learning. Consistent with this view, Loughman and Haslam (2018) suggested that a crucial element in the fight against mental health-related stigma is public communication that “emphasizes complexity over reduction, and plasticity over fixity”. A key lesson gained from research in social neuroscience is that categorization (or labeling), based on fundamental cues such as sex, race, and age, as well as other outwardly signs, or stereotypes (e.g., facial aesthetics and expression, or tattoos and dress style), may be activated in an automatic manner, enabling appraisal and evaluation of the subject, and shaping of the behavior by the perceiver (Macrae and Bodenhausen, 2000; Reihl et al., 2015; Comte et al., 2016). Therefore, examination of whether and how perceptions are subject to modulation (manipulation) would seem to be a worthwhile undertaking in the future.

The prejudice that leads to stigmatization and discrimination is considered to result from cognitive and affective responses to stereotypes where reflexive disgust is a common affective (defensive) emotion (Corrigan et al., 2001), followed by a rule-based process that derives from anticipated social interactions (Pryor et al., 2004). According to this schema, individuals (observers or bearers of stigma) can tweak their reflexive responses (disgust) to moderate their behavioral response (stigmatizing, discriminatory, courtesy or pity); in other words, although the initial emotional responses may be difficult to suppress (cf. Macrae and Bodenhausen, 2000), the expression of subsequent stigmatizing and related behaviors can be controlled. As considered in greater detail below, this view is supported by neuroimaging studies (e.g., by Krendl et al., 2006, 2012, 2013) but does not necessarily exclude the idea that individuals may be able to change their attitudes through a learning process.

As already alluded to, the language and concepts developed by social scientists and psychiatrists often differ; for example, while the former often refer to disgust as a trigger of stigma, the word rarely appears in the psychiatric literature on the subject. This is not surprising since stigma research in psychiatry has mostly focussed on the description of the types and extent of mental health-related stigma (e.g., Henderson et al., 2014; Roy et al., 2021), interventions aimed at stigma reduction within communities, and at policy building (e.g., Barbui et al., 2020; Greene et al., 2021; Roy et al., 2021). On the other hand, social neuroscience has contributed knowledge regarding the neuroanatomical correlates of the emotional and cognitive components of stigma. This is exemplified below by results from the work of Krendl et al.^5^:

- Consistent with previous functional neuroanatomical descriptions, studies demonstrated activation of the amygdala and insula in the evaluation and mediation of (automatic) emotional responses to aversive (disgust-inducing) stimuli, and activation of the anterior cingulate and dorso- and ventrolateral prefrontal cortex in higher order cognitive downregulation (control and inhibition) of emotional responses; the cortical regions showed higher latencies of response, suggesting that the emotional reactions precede cognitive regulation (Krendl et al., 2006, 2012, 2013). Krendl et al. (2012) also found activation of the right inferior frontal gyrus (IFG), an area that contributes to social cognition by inhibiting biased responses. In a subsequent study, Krendl (2016) reported on the additional involvement of the ventral striatum and parahippocampal gyrus in the perception and evaluation of negatively stigmatized persons.
- The neural networks involved in evaluating targets and regulating stigmatizing responses are smaller than those involved in general emotion regulation in which visuospatial processing may play an important additional role (Krendl et al., 2012). Notably, data from the latter study suggested that individuals with higher levels of negative bias require greater regulatory effort to reduce that bias. Further, the authors remarked on the possibility that individuals may engage cortical areas differently, depending on whether they feel compelled to suppress stigmatizing behavior or if they can freely express any prejudice they may hold against the stigmatized target.
- Examination of the question of which neural networks are engaged when an observer perceives a stigma to be controllable (target of stigma is perceived as being “responsible” for their condition) *vs*. uncontrollable revealed that the medial prefrontal cortex (implicated in intentionality) is more activated when the stigmatized person’s condition is perceived as self-controllable; in contrast, affective regions such as the insula (involved in attitude-formation) are more responsive when the stigmatized person is not perceived as being responsible for their existing condition (Krendl et al., 2013).
- The results of a study in which evoked potential responses were measured while subjects were exposed to pictures of individuals in negative, but non-stigmatized, circumstances, showed that stigmatized conditions generate faster, more prominent, robust and sustained responses than images of persons in non-stigmatized conditions; the sustained responses suggest that cognitive control mechanisms may be insufficient to negate the strong affective responses elicited by the stigmatized images (Krendl et al., 2017).
- while the affective response to persons experiencing stigmatizing circumstances may be largely independent of culture, they may be moderated by cultural bonds between the perceiver and the stigmatized (Krendl, 2016).

Since none of the above work was conducted in persons with mental health illness, it would be of interest to ask,

Are the above-described neuroanatomical substrates generalizable to stigma associated with mental health?

Stigma is usually only considered from the perspective of negative attitudes towards persons with attributes that do not conform with the expected norm. With one exception (Decety and Sommerville, 2003), the question of how positive mentalizing might influence stigmatizing behavior seems to have escaped the attention of neuroscientists. Given that empathy is a key element in patient-doctor relationships and coping and recovery from illness, insight into this issue could have practical implications for clinical practice.

The issue of empathy was examined in a recent study by Shin et al. (2020) who exploited the “self-other” concept embodied in the theory of mind^6^. These authors examined whether the identification of the self with illness in another elicits empathetic concern for the welfare of the other. Study participants (healthy male and female adults, mean age 22.8 ± 2.1 years) were asked to rate the value of supportive (caring) messages targeted at the other, from either the perspective of self (observer) or from the adopted perspective of either the physically or mentally ill person; participants underwent functional magnetic resonance imaging (fMRI) during the experiment. Participants identified closely with the physically ill when interpreting the supportive messages; this was not the case when participants observed the mentally ill from outside (self) or from within (as the patient themselves). The ratings correlated positively with participants’ disposition towards concern for the physically ill, and negatively with their stigma toward mentally illness. Interestingly, the fMRI scans revealed that confrontation with physical illness leads to greater activity in the ventromedial prefrontal cortex and superior frontal gyrus, whereas the dorsal anterior cingulate and anterior insula were more active when mental illness is encountered. The results of this study may be interpreted to imply a biological basis for the bias individuals apply when faced with persons bearing characteristics that can be identified or empathized with—in this case, physical discomfort or pain which most of us have experienced at some point in our lives^7^. But even if such bias is in-built, the question,

Are ill persons less deserving of empathy, care, support, and respect simply because they do not fit into a preconceived or expected “norm”?

is *not* a subject of debate, neither from the perspective of universally accepted Human Rights nor with respect to moral and ethical standards.

Shin et al. (2020) acknowledged that their participant cohort (relatively young students that were likely to have mindsets different from less educated or older members of the population) may have exerted some inhibitory cognitive control over any underlying prejudices (cf. Stull et al., 2013). Nevertheless, given that the brain structures engaged in the specific paradigm used by Shin et al. (2020) were mostly identical to those activated by other stigmatized scenarios (e.g., unattractiveness, overweight, transexual, piercings; alcohol and substance use or homelessness; homelessness due to own blame), it appears that there is a default set of neuroanatomical mediators of stigma, irrespective of the nature of the stigmatizing condition. On the other hand, still begging is the question,

Where and how might stigmatizing conditions be compared and calibrated so that the perceiver can make graded responses to different situations, for example physical vs. mental illness?

Unlike physical disabilities and states of obesity, for example, mental illness cannot be easily discerned from a person’s facial or other external cues and rather depends on the recognition of a behavior that deviates from the expected norm. Assuming that similar neurobiological substrates are engaged in the differentiation of “them” from “us”, lessons might be learnt from studies about how facial features may shape biased perception. Based on a review of the literature, Bagnis et al. (2019) developed a dynamic and interactive model to explain “social vision” (categorization) and intergroup bias. This model implies “recursive and dynamic interactions” between distant brain regions and proposes that “the reciprocal exchange of sensory evidence and prediction biases” is eventually balanced such that social perception is stabilized. Consideration of such a framework may be instructive in attempts to understand the neurobiological basis of stigmatizing attitudes towards persons with mental health issues, but also of how they develop self-stigma, and of how an individual’s stigmatizing attitudes may change with time.

Affective responses are set off in an automatic or reflexive manner upon confrontation with stimuli perceived to be threatening, generating fear (identifiable threats) or anxiety (non-specific threats). Although these responses may be programmed by genetic mechanisms,

genetics is not a tenable excuse for stigma since behaviors are subject to bi-directional modification through gene X environment interactions (epigenetics).

In fact, there is now ample evidence that epigenetic mechanisms underpin learning. At the same time, there is growing consensus that epigenetic plasticity (reversible and *de novo* epigenetic programming) facilitates the (theoretically *ad infinitum*) need to generate new and beneficial behavioral strategies. Accordingly, epigenetic plasticity may help explain the evolution of individual and societal attitudes towards persons once labeled as “different”.

As discussed before, emotions are subject to top-down regulation by cognitive processes. Studies by Krendl (see citations above), Comte et al. (2016), and Shin et al. (2020) highlighted the role of some key cortical and sub-cortical structures in the conscious detection of “conflict error awareness” (insula), feelings of disgust (amygdala), empathy (insula), response selection (anterior cingulate cortex), and in the prediction, attribution and memory of value, working memory and decision-making (prefrontal cortex). Here, the functions associated with these brain areas are necessarily over-simplified not only because studies of their involvement in stigma are few, but also because of the feedforward and feedback connections between them and other brain regions such as the visual and auditory cortices, hippocampus (learning and memory) and ventral striatum (motivation and reward). Although neuroimaging studies, coupled with monitoring of normal and pathological behaviors, are making strides in advancing our knowledge of functional networks and computational processing in the brain, they are far from complete. There remain opportunities to

explore brain regions and connectivity in the context of “self-other” interactions, and therefore help the development objective measures of the efficacy of behavioral interventions to reduce stigma.

We previously referred to the idea that stigma develops progressively from early childhood. At the same time, the individual is exposed to new experiences (political, racial, health etc) throughout life that may create new worldviews, resulting in new biases and prejudices. It is plausible that new experiences superimpose their effects on existing attitudes that then emerge as stronger or more complex stigmas—behavioral adaptations that depend on both, reinforcement and neuroplasticity (including re-connectivity), such as is observed during learning and memory. Accordingly, one might ask

If stigma involves learning and memory, can at least some of its components be erased, e.g., through behavioral therapy or education?

The above question does not dismiss the importance of the emotional component of psychotherapy. In fact, as previously mentioned, emotion, cognition (e.g., learning and memory) as well as behaviors are tightly coupled; it therefore follows that psychotherapy targets both emotions and cognition and subsequent behavioral responses.

Study designs aimed at examining the neurobiological mechanisms contributing to any form of stigma need to control for a variety of moderators. Besides socioeconomic, educational, and psychosocial (contact or support) factors, momentary experiences, and gender also need to be considered. Moreover, although stigma is a universal phenomenon, local traditions and cultural beliefs play an important role in determining stigmatizing behavior and its enactment. That stigma varies within communities and across regions is illustrated by data from a recent study in which the distribution and determinants of stigma around mental health were mapped in two provinces (Sofala and Manica) in central Mozambique (Zhang et al., 2019). The authors observed that higher levels of stigmatizing attitudes are held by persons aged 18–24, males, divorcees and widows/ers); low levels of education and wealth, urban life, as well as lack of religious beliefs are also associated with higher levels of stigmatizing attitudes towards persons with mental health problems.

There is strong public interest regarding the inner workings of the brain and of health in general. As neuroscientists, we are duty-bound to share our knowledge and to increase public awareness of advances in brain science. At the same time, and as already mentioned, it is important to be aware that, current educational programs aimed at providing the public with fundamental knowledge about the biological basis of mental illness may not have a sustained stigma-reducing effect (Loughman and Haslam, 2018; Lebowitz, 2019; Walsh and Foster, 2021). Given this, and in light of the “them” and “us” phenomenon, the organic nature of mental illness, and the interactions between emotional and cognitive behavior (e.g., learning, memory, and emotional and cognitive regulation and flexibility),

public education campaigns must be regularly reviewed and adapted for style and content. Confidence in the power of education as an anti-stigma intervention will depend on objective and statistically valid outcome measures. Lastly, reinforcement (renewed campaigns) and maximizing outreach are crucial to uptake and sustainability of actions involving education of lay audiences.

Finally, neuroscientific investigations could focus on the mechanisms through which narratives or storytelling by persons who have recovered from mental illness (i.e., persons with lived experience; Steffen, 1997) reduces shame among participants in peer-support groups as well as in anti-stigma campaigns (Roe et al., 2020). Questions that could be asked include,

- Which neural substrates (brain areas and connections), are activated or inhibited in narrators and listeners? In which sequence do these activations/inhibitions occur? Do narrators “relive” their experiences while storytelling?
- How are any observable changes in neural connectivity maintained and can these be reversed or perturbed?
- Do more stories (similar experiences or health conditions) have a reinforcing effect?

## Concluding Statement

This short article aims to encourage further research into the neurobiological substrates, pathways and mechanisms that underlie stigmatizing behavior. To that end, we sought to suggest questions worthy of future investigation in the hope of bridging traditional gaps between clinical behavioral neuroscience and social neurosciences. Most of the questions posed relate to anxiety/fear, learning and decision-making behaviors. It should be emphasized however that, while fear and anxiety are evolutionarily conserved behaviors that are crucial for survival, their inefficient regulation can result in harmful (stigmatizing) attitudes and actions (discrimination). Lastly, it should be noted that the implications of research in this area are broad: stigmatizing and discriminatory practices not only impinge on the rights of others but also extend to the economic costs of maintaining global health (Maj, 2011; Wahl, 2012).

## Author Contributions

OA conceived the review and surveyed the literature. Both authors wrote the article. All authors contributed to the article and approved the submitted version.

## Conflict of Interest

The authors declare that the research was conducted in the absence of any commercial or financial relationships that could be construed as a potential conflict of interest.

## Publisher’s Note

All claims expressed in this article are solely those of the authors and do not necessarily represent those of their affiliated organizations, or those of the publisher, the editors and the reviewers. Any product that may be evaluated in this article, or claim that may be made by its manufacturer, is not guaranteed or endorsed by the publisher.

## Footnotes

^1^Although stigma is cross-sectoral, this essay mainly considers the subject from the perspective of mental health, reflecting the authors’ specific interests.

^2^The report declared that “the single most important barrier to overcome in the community is the stigma and associated discrimination toward persons suffering from mental and behavioral disorders”. Despite incremental improvements, stigma remains a problem of sizable proportions.

^3^Throughout this essay, we consider stigma as a phenomenon experienced by people with characteristics that make them stand out as different from most of the population in which they live. While it is true that certain illnesses such as diabetes are viewed in a better light than mental disorders, there are conditions such as human immune deficiency (HIV)/acquired immune deficiency syndrome (AIDS) that receive a far more hostile response than mental diseases in terms of stigma (Robinson et al., 2019). Another example of this comes from the COVID-19 pandemic where those infected with the virus often faced stigmatizing behaviors that, in some cases, even escalated into xenophobic behavior (Gover et al., 2020). As with HIV-positive and AIDS persons, those with COVID-19 are often considered incurable; apart from being socially isolated, the stigma attached to COVID-19 infection potentially increases the risk of health deterioration.

^4^The word “condition” was chosen here instead of the more common (and correct) term “disorder” which could be perceived as part of a negative label. Mental Health Europe (https://www.mhe-sme.org) recommends: (i) being careful with labels, not referring to people as their condition; (ii) speaking about a person’s experience, rather than their symptoms; and (iii) to avoid potentially harmful clichés. These views are echoed in a recent instructive commentary by the directors of three of the US National Institutes of Health that cover mental illness and substance use disorders (Volkow et al., 2021).

^5^Krendl and colleagues’ work has of course been preceded by investigations by other authors but has also been confirmed or extended in other studies—for a comprehensive review, see Amodio and Cikara (2021). Readers interested in further details on the functional neuroanatomy of implicit and explicit bias may also refer to Reihl et al. (2015).

^6^Theory of mind refers to the ability to mind-read, mentalize, explain and predict the beliefs, desires, intentions, or actions of others.

^7^Readers should be aware of possible bias in the interpretation of the results from Shin et al. (2020): whereas the mental disorders presented (schizophrenia, depression, paranoid disorder, and OCD) are all highly stigmatized, the physical diseases presented (leukaemia, cancer, diabetes, and arthritis) are less-stigmatized as compared to some other physical conditions such as HIV positivity.
